# Infection Control among Healthcare Workers and Management of a Scabies Outbreak in a Large Italian University Hospital

**DOI:** 10.3390/jcm12113830

**Published:** 2023-06-02

**Authors:** Stefania Sponselli, Luigi De Maria, Antonio Caputi, Pasquale Stefanizzi, Francesco Paolo Bianchi, Giuseppe Delvecchio, Caterina Foti, Paolo Romita, Francesca Ambrogio, Silvia Zagaria, Gianmarco Giannelli, Silvio Tafuri, Luigi Vimercati

**Affiliations:** 1 Interdisciplinary Department of Medicine, University of Bari, 70124 Bari, Italy; stefania.sponselli@uniba.it (S.S.); luigi.demaria@uniba.it (L.D.M.); antonio.caputi@uniba.it (A.C.); pasquale.stefanizzi@uniba.it (P.S.); frapabi@gmail.com (F.P.B.); giuseppe.delvecchio1@uniba.it (G.D.); silvia.zagaria@uniba.it (S.Z.); gianmarco.giannelli@uniba.it (G.G.); silvio.tafuri@uniba.it (S.T.); 2 Department of Biomedical Science and Human Oncology, University of Bari, 70124 Bari, Italy; caterina.foti@uniba.it (C.F.); paolo.romita@uniba.it (P.R.); dottambrogiofrancesca@gmail.com (F.A.)

**Keywords:** scabies, outbreak, prevention protocol, healthcare workers

## Abstract

This retrospective observational study describes the results of an ad-hoc designated prevention protocol aimed at containing the spread of the scabies infestation among healthcare workers (HCWs) of a large University Hospital in Italy. The outbreak started on October 2022 and a preventive protocol was set up thanks to a multidisciplinary approach. HCWs at high scabies risk were defined as subjects working in Operative Units with a scabies prevalence higher than 2%, close contacts of a confirmed case of scabies, or HCWs with signs and symptoms of the disease. All cases at high scabies risk underwent a dermatological examination, and the infested HCWs were suspended from work until definitive healing. Mass drug administration was established for all HCWs working in Operative Units with a scabies prevalence higher than 2%. Until March 2023, out of 183 screening dermatological examinations, 21 (11.5%) were diagnostic for scabies. Between 11 October 2022 (date of the first diagnosed scabies case) and 6 March 2023 (the end of incubation period related to the last case detected), the frequency of scabies was 0.35% (21 scabies cases/6000 HCWs). The duration of the outbreak in our hospital was 14.7 weeks. Statistical analysis shows a significant association between scabies and being a nurse and having an allergy to dust mites. We obtained a low frequency of scabies infection, limiting the duration of the outbreak and the related economic burden.

## 1. Introduction

Scabies is one of the most common skin infestations in the world caused by the human itch mite, Sarcoptes scabiei var. hominis. It is estimated to affect more than 200 million people worldwide, with a prevalence ranging between 0.2% and 71% [1]. Scabies is endemic in underdeveloped and developing countries, although it could be considered as a re-emerging infectious disease also in developed community settings, especially in crowded conditions [2,3,4,5,6]. The most common sites of scabies outbreaks are nursing homes, prisons, or childcare facilities. Nosocomial scabies outbreaks could represent a major public health problem, and this is accentuated by the high costs it places on healthcare systems [1,7].

Scabies is transmitted by direct and prolonged skin-to-skin contact with infected individuals, even if asymptomatic. The mite’s evolutionary cycle takes place entirely in man: after mating, the adult male remains on the skin while the female burrows into the epidermis, where she usually lives for 30 days, laying her eggs. After a few days, the eggs hatch and the larvae invade the skin, triggering a local inflammatory response with a rash (and the concomitant intense itching) being the most common symptom. Usually, in a primary infestation, this reaction occurs after about 4 weeks by the contact with an infested individual [8]. The incubation period of scabies is estimated between 2 and 6 weeks, except for reinfections, where it is shorter [9].

When scabies affects immunocompromised, elderly, or debilitated people, it can lead to a more severe infestation called Norwegian or crusted scabies. This kind of infestation is more contagious because of the large number of mites colonizing a patient’s skin (up to 2 million). People affected by crusted scabies can easily spread infestation either through short skin-to-skin direct contact or indirect contact with clothes, beds, or infested fomites [7].

Scabies outbreaks can be more frequent in healthcare settings, due to the clinical peculiarities of patients (e.g., physical or mental disability, advanced age, immunodeficiency), the duration of hospitalizations, and the direct physical contact necessary for assistance. Most of the outbreaks in healthcare settings are caused by unrecognized Norwegian scabies in hospitalized patients [10,11,12,13,14]. Moreover, the diagnostic delay may underestimate the prevalence of scabies in these contexts [15]. Usually, a lot of patients and healthcare workers (HCWs) are exposed to scabies, both in underdeveloped and developed countries. To date, there are no univocal recommendations on the mass drug administration (MDA) or measures to prevent the transmission of scabies in close contacts [16]. Similar to what was observed during the COVID-19 pandemic, the implementation of preventive protocols, aimed at a correct management of outbreaks, is crucial to limit the spread of infectious diseases among HCWs in healthcare settings [17]. 

The aim of this retrospective observational study is to analyze a scabies outbreak in a large University Hospital in the South of Italy and to describe the results of an ad hoc designated prevention protocol aimed at containing the spread of the infection among HCWs.

## 2. Materials and Methods

### 2.1. Setting and Participants

The outbreak started on 11 October 2022 with a cluster of scabies cases in the Operative Unit (UO) of Internal Medicine (division F.). The observation period was between 11 October 2022 and 6 March 2023, considering the first case of scabies and the end of incubation period related to the last case detected (6 weeks starting from 22 January 2023). The ad hoc designated preventive protocol was applied for all healthcare workers (HCWs, about 6000) working at the University Hospital of Bari, Apulia, Southern Italy. Ethical approval was not necessary because all medical examinations were performed according to Italian law on the protection of workers exposed to occupational risks (D.Lgs. 81/2008) and with the principles of the Declaration of Helsinki, with scientific methods and for scientific purposes.

### 2.2. Case Definitions

According to the 2020 International Alliance for the Control of Scabies (IACS) Consensus Criteria for the diagnosis of scabies, a “confirmed” case of scabies (level A) is defined as a patient who presents mites or mite products (e.g., eggs, faeces) visible by using a high-powered device and/or dermoscopy and/or light microscopy of skin samples [18]. If mites or their products are not visible and other differential diagnosis are less likely than scabies, the diagnosis of scabies could also be “clinical” (level B) when the patient presents burrows and/or typical skin lesions in the male genital area and/or in a typical distribution, with at least two anamnestic features (e.g., itch, close contact with a scabies case). Diagnosis of “suspected” scabies (level C) can be made if the patient presents typical skin lesions in a typical distribution, without any anamnestic features, or if he presents atypical skin lesions in atypical distribution, with at least two anamnestic features. 

According to the European Guideline for the management of scabies, a scabies case could be considered cured if there are no signs or symptoms of scabies (e.g., absence of active skin lesions or nocturnal itching) within one week of starting therapy [19].

### 2.3. Epidemiologic Investigation and Notification System

According to Italian law, all suspected or confirmed cases of scabies must be notified to the competent health authority [20]. In this study, the epidemiological investigation was conducted by the UO of Hygiene and a report was also sent to the UO of Occupational Medicine to temporarily remove HCWs from work, according to the preventive protocol.

### 2.4. Prevention and Protection Measures

**Figure 1:**
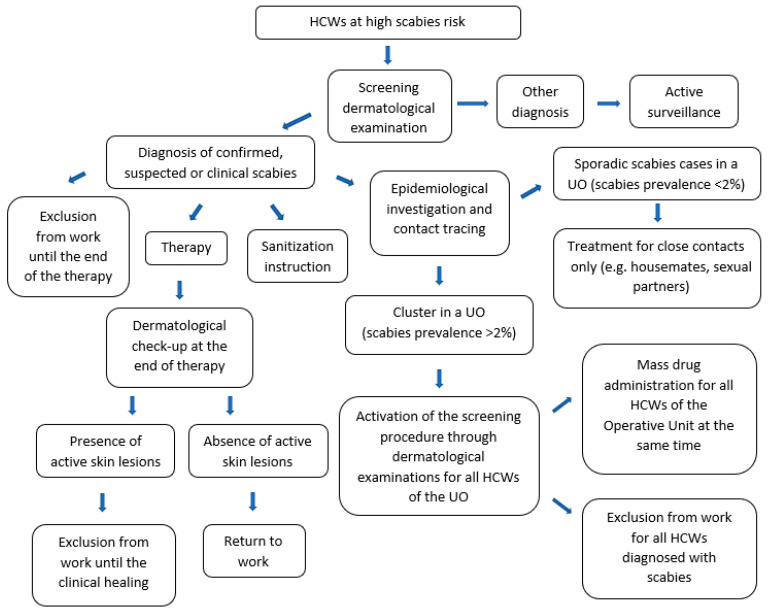
Prevention protocol for the management of scabies hospital outbreaks.

In Italy, the management of scabies cases is still based on the indications given in the circular of the Ministry of Health of 13 March 1998, which requires isolation of cases for 24 h from the start of treatment [21]. In this study, the prevention protocol was designated by the UO of Occupational Medicine to limit the spread of scabies among HCWs (Figure 1). As for all transmissible infectious diseases, the protocol was adapted considering the peculiar healthcare setting in order to protect the health of both personnel and frail patients at high risk of comorbidity, similar to what was observed during the COVID-19 pandemic [22,23].

First, a risk assessment was carried out, defining HCWs at high scabies risk as subjects working in UOs with a scabies prevalence higher than 2%, close contacts of a confirmed case of scabies, or HCWs with signs and symptoms of the disease (e.g., generalized itch, intense at night; skin lesions disseminated on wrists, extensor surfaces of the upper and lower limbs, fingers or interdigital spaces, breasts, genitalia, buttocks, and/or waist).

According to the protocol, all cases of HCWs at high scabies risk were subject to active surveillance and underwent a screening dermatological examination at the UO of Dermatology. In particular, all HCWs were informed on self-monitoring of scabies signs and symptoms and invited to report their eventual onset to the UO of Occupational Medicine.

All scabies cases were treated by applying topical Permethrin 5% cream or Benzyl benzoate 25%. The latter was used by applying the lotion all over the body for 10 h for three consecutive days and repeating the treatment after one week, according to the literature data [24]. Regarding Permethrin, although the European Guideline for scabies recommend applying the cream all over the body for 10–12 h and repeating the treatment after 1 or 2 weeks, in this study, it was used for two consecutive days, repeating the treatment for a single day after 1 week. This therapeutic protocol was established by dermatologist specialists of the UO of Dermatology on the basis of their clinical expertise, the good results obtained, and years of experiences in outpatient dermatological visits. Therapy-refractory cases were also treated with oral Ivermectin. It was also recommended to treat close contacts (e.g., people living in the same house or sharing the same bed) at the same time [19]. 

Although national and international recommendations suggest a 24-h absence from work from the start of therapy, a rapid and more aggressive response to institutional outbreaks is equally recommended [21,25]. Therefore, our protocol established that all infested HCWs were suspended from work for the whole duration of treatment, until definitive healing. At the end of treatment, all HCWs infested underwent a medical check-up at UOs of Dermatology and Occupational Medicine in order to certify the absence of active skin lesions after at least one week from the end of treatment and to allow the return to work. 

All cases were also instructed to wash sheets, blankets, and clothes at 60 °C or more, or to close them into a plastic bag or cellophane, without using them for a week to avoid reinfestations. 

All Directors of Operating Units with reported scabies cases were also requested to identify all possible contacts among HCWs or patients and to ensure the cleaning of all surfaces or furnishings (e.g., rooms, bathrooms, padded furniture) using a vacuum cleaner, or to close all non-washable items in a plastic bag or a cellophane for a few days.

According to the European Guideline for the management of scabies, in case of epidemical outbreaks, MDA was extended to all contacts, even if not close (level of evidence Ib; grade A recommendation) [19,26]. As reported in the literature, MDA is recommended when the prevalence of scabies is higher than 10% and is not recommended when the prevalence is lower than 2%. To date, there are no univocal recommendations on the control strategy when the prevalence of scabies is between 2% and 10% [27]. In our protocol, a single-dose permethrin-based MDA strategy was used for all HCWs working in UOs with a scabies prevalence higher than 2%. In particular, a single dose of 5% permethrin was administered to all HCWs on the same day.

### 2.5. Statistical Analysis

Continuous variables are reported as mean ± standard deviation (SD) and range, and categorical variables as proportions. The chi-square and the Fisher’s exact tests were used to compare proportions between groups. The Skewness and kurtosis test was conducted to evaluate the normality of the continuous variables; a normalization model was set for not normally distributed variables; and the t student for independent data test was performed to compare continuous variables between groups.

To analyse the determinants of scabies, a multivariate logistic regression model was built; the variables of age, sex (male vs. female), UOs (Internal Medicine F. vs. other), job category, BMI, smoke habit, use of alcohol, allergies, and chronic conditions were considered as determinants. The adjusted Odds Ratios (aORs) were calculated as well as 95%CIs. The Hosmer-Lemeshow’s chi-squared test was used to evaluate the goodness-of-fit of the multivariate logistic regression model.

A two-sided *p*-value < 0.05 was considered an indicator of statistical significance for all tests. An anonymized data analysis was performed using the STATA MP17.0 software.

## 3. Results

### 3.1. Scabies Cases: Frequency and Distribution

Table 1 shows the time trend of the spread of the infection. Overall, scabies cases were observed in eight UOs: Internal Medicine divisions F. (*n* = 13) and B. (*n* = 1), Pneumology (*n* = 1), Emergency Medicine and Surgery (*n* = 1), Transfusion Medicine (*n* = 1), Pediatric Oncology (*n* = 1), Rheumatology (*n* = 1), and Cardiac Surgery (*n* = 2).

In the observation period, 183 HCWs at high scabies risk were subjected to screening dermatological examinations: 161 (88%) were negative, 21 (11.5%) were found to be diagnostic for confirmed scabies (level A, IACS), and 1 (0.5%) was diagnosed for other parasitosis. Close contact treatment with topical Permethrin 5% cream was also indicated for 39 asymptomatic HCWs (21.3% of all dermatological examinations). 

Between 11 October 2022 (date of the first diagnosed scabies case) and 6 March 2023 (the end of incubation period related to the last case detected), the frequency of scabies in our hospital was 0.35% (21 scabies cases/6000 HCWs). Among scabies cases, there were eight physicians (five male, three female), seven nurses (two male, five female) and six other HCWs (four male, two female). The average age was 37.8 years, although it was lower in females than in males (33.9 years vs. 41.3 years) and in physicians than in other HCWs (29.75 years for physicians vs. 39.43 years for nurses vs. 46.5 years for other HCWs). Among scabies cases, all HCWs showed scabies burrows and typical itchy lesions in a typical distribution (e.g., wrists, interdigital spaces, genitalia, etc.). 

The average length of the infestation period was 31 days (range 8–48 days).

### 3.2. Characteristics of The Sample and Risk Factors of Scabies

The characteristics of the sample of HCWs at high risk, per group, (scabies, *n* = 21) vs. control (non-scabies, *n* = 162), are reported in Table 2.

The multivariate regression model showed a statistically significant association between the diagnosis of scabies and allergy to dust mites (aOR = 8.0; 95%CI = 1.1–58.3), allergy to other antigens (aOR = 0.05; 95%CI = 0.01–0.57), and being a nurse (aOR = 7.1; 95%CI = 1.7–29.9) or another HCW other than a physician (aOR = 9.1; 95%CI = 1.8–46.2). No associations were found between the diagnosis of scabies and age, sex, BMI, smoke habit, alcohol, diabetes, hypertension, and cardiovascular diseases (Table 3).

### 3.3. The Index Case and Outbreak Reconstruction

The index case (IC) was identified through epidemiological investigation carried out by the UO of Hygiene. IC was an elderly man hospitalized in the UO of Internal Medicine F. from 27 July to 17 August 2022. During the hospitalization, the patient showed itchy skin lesions and was initially diagnosed with a suspected allergic reaction to drugs. After two months, IC underwent several medical examinations at the same UO, complaining about the persistence of skin lesions and, finally, on 12 October, was directed to a dermatological examination, which allowed the two-months-delayed crusted scabies diagnosis. 

The first scabies case among HCWs (a male physician) was detected on 11 October 2022 in the same UO that had initially assisted the IC. In the following days, seven other cases were diagnosed (eight cases overall in the UO of Internal Medicine F.). The first scabies case in a UO other than that of the index case (UO of Pneumology) was detected on 24 October 2022. The epidemiological investigation revealed that the HCW had close contact with colleagues working in the UO of Internal Medicine F. (the two wards were in the same floor), who were later diagnosed with scabies (he did not take any treatment because he was off work and unaware of the hospital outbreak). 

According to the preventive protocol, due to the high number of scabies cases in the UO of Internal Medicine F. (scabies prevalence higher than 2%), all HCWs underwent a screening dermatological examination. Overall, from 9 November 2022 to 6 December 2022, 152 HCWs working in the Operative Unit of Internal Medicine F. were submitted to screening specialistic examination: 3 (2%) were diagnosed with scabies (2 of them shared the same house) and none of the remaining 149 HCWs (88%) showed signs or symptoms of scabies at the time of the examination. The preventive protocol also required MDA for all HCWs working in the same UO through the simultaneous application of a single dose of Permethrin 5% topical cream on the so-called “T-day” (day of treatment) established on 20 November 2022. 

**Figure 2:**
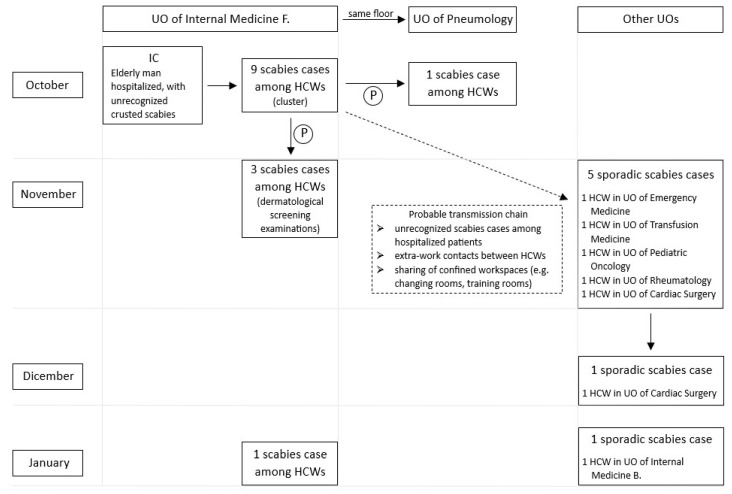
Outbreak reconstruction. Distribution of cases among HCWs of different UOs during the observation period. P: ad hoc preventive and protective protocol.

From the T-day to 22 January 2023, there were only few sporadic scabies cases in several different UOs (Figure 2).

## 4. Discussion

The prevalence of scabies in a healthcare setting could be underreported, often due to diagnostic delay and the presence of elderly immunocompromised in acute or long-term care facilities, with significant economic implications related to pharmaceutical costs, lost working days, or closures of hospital wards [15,28,29]. 

In our hospital, after the recognition of the scabies outbreak, a preventive protocol was urgently set up thanks to a multidisciplinary approach (cooperation between the Operative Units of Occupational Medicine, Hygiene, and Dermatology). Setting up a multidisciplinary team is the first recognized step towards an effective approach to the outbreak [30]. A crucial role within our protocol was also played by the massive information of patients and staff as well as by a careful risk assessment aimed at selecting the HCWs with high scabies risk to be sent to dermatological screening for any eventual therapeutic treatment. A HCW at high scabies risk was defined as a subject working in a UO with a scabies prevalence higher than 2%, a close contact of a confirmed case of scabies, or a HCW with signs and symptoms of the disease. This approach, which differentiates our protocol from those adopted in other similar experiences in the literature, has made it possible to contain the costs of MDA for all HCWs of the hospital (about 6000 HCWs), which, on the contrary, would have become necessary in the absence of a risk assessment and of an infection control strategy. 

Our protocol also provided stricter criteria for the suspension from work of HCWs diagnosed with scabies (the whole duration of the therapy and clinical recovery ascertained by medical examination before readmission to work) than national and international recommendations, which indicate suspension from work for only 24 h from the beginning of the therapy [21,26]. 

These preventive measures allowed us to obtain a low frequency of scabies (0.35%) in the University Hospital of Bari. The duration of our outbreak was short (14.7 weeks) and similar to the main duration of other described institutional outbreaks. Moreover, except for the Internal Medicine UO, the other UOs reported only sporadic cases, with no further clusters. This resulted in a lower impact in terms of work productivity. Supporting our results, the literature data on nosocomial scabies outbreaks showed that the average number of HCWs diagnosed with scabies per outbreak is 39 (range 6-278) and the average duration of outbreaks is 14.5 weeks (range 4–52 weeks) [28]. 

As regards the characteristics of scabies cases, our results are in line with the literature in showing a significant association between scabies infection and being a nurse [28]. Moreover, our study showed a significant association between scabies infection and having an allergy to dust mites. A possible explanation for this finding is that the immune reaction of subjects with a dust allergy is more intense and produces a more evident clinical outcome related to scabies infection (e.g., skin lesions) and, consequently, is easier to recognize. According to the literature data, our study also showed no statistically significant association between having chronic diseases (e.g., diabetes, hypertension, and cardiovascular diseases) and scabies [31]. Finally, a literature review by Dobner et al. showed an association between obesity and skin infection rates [32]. In contrast with this finding, our study showed no statistical associations between scabies diagnosis and BMI or age.

Our study has some limitations, including its retrospective design. The potential diagnostic delay, due to the extended incubation period (during which the asymptomatic subject could transmit the infection) and the individual variability of clinical manifestations of the infestation, could have led to an underestimation of scabies cases. Moreover, dermatological screening was carried out only for high risk HCWs, according to risk assessment and epidemiological investigation. On the one hand, this attitude could have led to an underdiagnosis but, on the other, it allowed us to save financial resources and time, which could be reserved for the treatment of cases and their clinical evaluation for readmission to work, and this represents one of the strengths of the study. In particular, in our study, the total cost of the scabies outbreak management was about €32,700, considering the economic cost due to sick leave among HCWs and the overall costs of screening dermatological examinations and pharmacological therapy for both scabies cases and contacts. The average cost of MDA for all HCWs would have been five times higher than the cost of the scabies outbreak in our study.

Finally, an important point that would further improve the management of scabies outbreaks in hospital settings is the strengthening of the interactions and the shared data network between hospitals and local health authorities. Concerning this, as reported in the literature, some symptomatic HCWs consult the general practitioner rather than the occupational health service or hospital dermatologists, generating a greater probability of misdiagnosis and compromising contact tracing procedures [33]. Nevertheless, coordinated actions to control scabies at a global level are crucial, since scabies outbreaks are likely to increase in the near future as a result of an ageing population and increasing population density in urban areas [27,34,35].

## 5. Conclusions

The specific approach to scabies outbreaks in healthcare settings has gained increasing importance in management and treatment guidelines, although there is no globally accepted strategy, and there are still different issues to be addressed. Within this context, our study shows our experience of how a nosocomial scabies outbreak was contained through the rapid implementation of prevention protocol and measures that allowed us to limit the spread of the infestation among HCWs, the duration of the outbreak, and the economic burden related, in particular, to HCWs absence from work.

Nevertheless, the strengthening of the interactions between the hospital and territorial care networks is necessary to guarantee a more optimal management of hospital outbreaks and to limit missing diagnoses. Further studies may increase our knowledge of the best approach to an emerging and recurrent issue in hospital institutions, and reviews of these outbreaks and their approach could also be used in working contexts other than the healthcare one.

## Figures and Tables

**Table 1:** Spreading of scabies outbreak in the observation period.

		Number of Cases of Confirmed or Suspected Scabies						
Month	Internal Medicine Division F.	Internal Medicine Division B.	Pneumology	Emergency Medicine and Surgery	Transfusion Medicine	Pediatric Oncology	Rheumatology	Cardiac Surgery
October 2022	9	-	1	-	-	-	-	-
November 2022	3	-	-	1	1	1	1	1
December 2022	-	-	-	-	-	-	-	1
January 2023	1	1	-	-	-	-	-	-
February 2023	-	-	-	-	-	-	-	-
March 2023	-	-	-	-	-	-	-	-

**Table 2:** Characteristics of the sample, per group.

Variable	Scabei (*n* = 21)	Control (*n* = 162)	Total (*n* = 183)	*p*-Value
Age; mean ± SD (range)	37.81 ± 12.56 (24–65)	39.29 ± 13.07 (23–70)	39.12 ± 12.99 (23–70)	0.493
Males; *n* (%)	11 (52.38)	60 (37.04)	71 (38.80)	0.175
Internal Medicine F. Unit; *n* (%)	13 (61.90)	157 (96.91)	170 (92.90)	<0.0001
Job category; *n* (%) • Physician • Nurse • Other	8 (38.10) 7 (33.33) 6 (28.57)	106 (65.43) 29 (17.90) 27 (16.67)	114 (62.30) 36 (19.67) 33 (18.03)	0.051
BMI; mean ± SD (range)	24.34 ± 3.03 (19.56–29.23)	24.11 ± 3.82 (17.04–42.83)	24.14 ± 3.73 (17.04–42.82)	0.653
Smoke; *n* (%)	6 (28.57)	42 (25.93)	48 (26.23)	0.795
Alcohol; *n* (%)	6 (28.57)	71 (43.83)	77 (42.08)	0.183
Allergy: • dust mites; *n* (%) • other; *n* (%)	3 (14.29) 1 (4.76)	18 (11.11) 40 (24.69)	21 (11.48) 41 (22.40)	0.668 0.039
Diabetes; *n* (%)	1 (4.76)	5 (3.09)	6 (3.28)	0.685
Hypertension; *n* (%)	2 (9.52)	18 (11.11)	20 (10.93)	0.826
Cardiovascular diseases; *n* (%)	3 (14.29)	7 (4.32)	10 (5.46)	0.059
Lung diseases; *n* (%)	8 (4.94)	0 (0.00)	8 (4.37)	0.298
Nephropathies; *n* (%)	3 (1.85)	0 (0.00)	3 (1.64)	0.529
Steroids; *n* (%)	8 (4.94)	0 (0.00)	8 (4.37)	0.298
Immunosuppression/depression; *n* (%)	1 (4.76)	2 (1.23)	3 (1.64)	0.231
Other therapies; *n* (%)	9 (42.86)	55 (33.95)	64 (34.97)	0.421

**Table 3:** Analysis of determinants of diagnosis of scabies through univariate and multivariate logistic regressions.

Determinant	Univariate		Multivariate	
	OR (95%CI)	*p*-Value	aOR (95%CI)	*p*-Value
Age	0.99 (0.96–1.03)	0.623	0.96 (0.91–1.02)	0.138
Sex (male vs. female)	1.87 (0.75–4.66)	0.179	3.12 (0.84–11.65)	0.089
Job category • nurse vs. physicians • other vs. physicians	3.20 (1.07–9.55) 2.94 (0.94–9.20)	0.037 0.063	7.05 (1.66–29.90) 9.05 (1.79–46.24)	0.008 0.008
BMI	1.02 (0.90–1.14)	0.793	0.95 (0.80–1.12)	0.538
Smoke habit	1.14 (0.42–3.14)	0.796	0.66 (0.20–2.17)	0.494
Alcohol	0.51 (0.19–1.39)	0.189	0.96 (0.29–3.19)	0.941
Allergies • Dust mites • Other	1.33 (0.36–4.98) 0.15 (0.02–1.17)	0.071 <0.0001	8.02 (1.10–58.29) 0.05 (0.01–0.57)	0.040 0.016
Diabetes	1.57 (0.17–14.13)	0.687	1.30 (0.10–17.60)	0.843
Hypertension	0.84 (0.18–3.92)	0.827	0.38 (0.04–4.10)	0.426
Cardiovascular diseases	3.69 (0.88–15.54)	0.075	1.63 (0.26–10.10)	0.599
Other therapies	1.46 (0.58–3.67)	0.423	2.19 (0.61–7.91)	0.230
Immunosuppression/depression; *n* (%)	8.05 (0.48–133.78)	0.146	1.64 (0.10–27.46)	0.732

## Data Availability

The data presented in this study are available on request from the corresponding author.
